# Using proteomics and single-cell sequencing to analyze the pathogenesis of recurrent implantation failure associated with uterine natural killer cells

**DOI:** 10.1007/s00404-025-08074-8

**Published:** 2025-06-13

**Authors:** Yu-Qin Hu, Xin-Xian Zhang, Ting-Ting Zhao, Xiao-Hua Wu

**Affiliations:** 1 Hebei Medical University, 361 East Zhongshan Road, Shijiazhuang, Hebei China; 2 Reproductive Medicine Center, The Fourth Hospital of Shijiazhuang, Hebei Medical University, Hebei, China

**Keywords:** Recurrent implantation failure, Proteomics, Single-cell sequencing, NK cells

## Abstract

**Problem:**

Recurrent implantation failure (RIF) affects about 10% of infertility patients and may involve mid-luteal phase endometrial natural killer (NK) cells. The pathogenesis of NK cells in RIF remains unclear. Method of study: Our study integrated proteomics data from endometrial tissues of six RIF patients and six controls, with single-cell sequencing insights.

**Results:**

Our proteomics analysis identified 1366 differentially expressed proteins (DEPs) between RIF and control groups, highlighting alterations in cellular processes, such as cytoplasmic translation and mRNA processing. Functional enrichment analysis revealed significant associations with pathways involved in amyotrophic lateral sclerosis and proteasomes. The DEPs were transformed into differentially expressed genes1 (DEGs1) by ID-transformation. 33 candidate genes were detected when ID-transformed 1210 DEGs1 were intersected with 752 DEGs2 from NK cells. After that, the proteomics sequencing data validation showed that the expression of AMPD3, H6PD, and PAK2 was consistent and significantly different from the GSE111974 dataset and classified as crucial genes. In addition, analysis of single-cell sequencing data annotated fibroblast-like stromal cells, NK cells, T cells, and endothelial cells, and these three essential genes showed that they were expressed in NK cells. Crossing the signaling pathways of key genes with those enriched for DEPs yielded the ‘*Escherichia coli* infection’ possibly associated with RIF. Finally, the transcription factor *HR* had a strong regulatory effect on the *PAK2*.

**Conclusion:**

Finally, identifying three key genes (*AMPD3*, *H6PD* and *PAK2*) associated with RIF and the regulatory solid roles of HR and PAK2 provided a basis for understanding the molecular mechanism of RIF. Our findings may pave the way for developing targeted therapies to improve pregnancy outcomes in patients with RIF.

**Supplementary Information:**

The online version contains supplementary material available at 10.1007/s00404-025-08074-8.

## Introduction

Globally, it is estimated that 8–12% of couples experience infertility, a condition that has been significantly alleviated by the advent of Assisted Reproductive Technology (ART). Despite the widespread application of ART for over four decades, post-in vitro fertilization-embryo transfer (IVF-ET) treatments result in failure to conceive for more than 60% of female patients following their initial embryo transfer. Moreover, nearly 20% of these patients endure unexplained recurrent implantation failures (RIF) and recurrent pregnancy loss (RPL) [1]. RIF is a huge challenge for reproductive doctors [2]. This underscores the pressing need to elucidate the etiology of RIF and develop more effective therapeutic interventions aimed at enhancing pregnancy rates within the domain of assisted reproduction.

There is no consensus on the definition of RIF. Currently, some studies suggest that RIF refers to individuals who have failed three or more embryo transfers or the transfer of 4 high-scoring cleavage-stage embryos or 3 or more high-scoring blastocysts in assisted reproductive treatments [3]. In conjunction with previous research, the present research defines as follows: RIF is characterized by the inability to detect a positive β-HCG in the blood or urine after a minimum of three consecutive embryo transfers of morphologically selected, good-quality embryos in patients under the age of 35 who are undergoing in IVF-ET procedures [4]. Approximately 10% of patients undergoing infertility treatment are affected by RIF [5]. Risk factors associated with RIF encompass a spectrum of influences, including advanced maternal age, tobacco use, and psychological stress, among others. Additionally, aberrant endometrial conditions, such as chronic endometritis, asynchrony between the embryo and endometrium, endometrial injury, and alterations in the immune system, are recognized as contributing factors to RIF [6]. Studies have reported that two-thirds of RIF cases is caused by abnormal endometrial receptivity, and immune cells play a significant role in endometrial receptivity [7]. Studies have demonstrated that within the endometrial immune cell population, uterine natural killer cells (uNK) exhibit the most robust proliferative response during the secretory phase of the menstrual cycle. Furthermore, during early pregnancy, uNK cells constitute over 70% of decidual leukocytes, highlighting their numerical predominance at the maternal–fetal interface [8]. NK cells are pivotal immune constituents within the female reproductive system, playing a crucial role in pregnancy-related immunity against infections, trophoblast invasion, and the remodeling of uterine spiral arteries, all of which are essential for maintaining a healthy pregnancy [9]. Therefore, focusing on the role of uNK cells in the mid-luteal phase in RIF is of significant importance.

Proteomics is a technology to study the composition and function of proteins in organisms. By analyzing types, quantities, and modifications of proteins expressed in cells or tissues, proteomics reveals the mechanism of action of proteins in cells and explores the biological processes of various life activitiess [10]. Combining single-cell RNA sequencing (scRNA-seq) with advanced data science has become a valuable technique for exploring transcriptional profiles related to disease progression. It enables the mapping of the composition and changes of various cell subpopulations within complex tissues, revealing dynamic transitions in cellular states that transiently or permanently control pathological phenotypes during the gradual development of diseases [11, 12].

In this study, we conducted a proteomic analysis of endometrial tissues collected during the mid-luteal phase from six patients diagnosed with RIF and six control subjects who were treated at our facility. Additionally, we integrated single-cell sequencing data obtained from reputable databases. The aim of this comprehensive approach was to elucidate the pathogenic mechanisms associated with uNK cells in the context of RIF.

## Materials and methods

### Protocol approval and patient consent

The study protocol received approval from the Institutional Review Board of Shijiazhuang’s Fourth Hospital (No. 20200062), and written informed consent was obtained from all participating patients. The above information appears in Supplementary Table 1.

### Recruitment

Two groups of female participants were selected for this study: those who experienced RIF (no biochemical pregnancy after ≥ 3 consecutive embryo transfers with a total of ≥ 4 good-quality embryos and re-transplantation required; *n* = 6), and those without RIF serving as controls (*n* = 6). The eligibility criteria stipulated that all participants must be under 35 years of age, possess normal ovarian reserve, and should not have undergone any targeted treatment for immune dysregulation during the cycle of endometrial biopsy. The exclusion criteria included recent use of contraceptives; hormonal or metabolic disorders, such as polycystic ovary syndrome, diabetes mellitus, type 2 diabetes, and hypothyroidism; genetic abnormalities; severe cases of adenomyosis or endometriosis; untreated hydrosalpinx; moderate to severe intrauterine adhesions; uterine malformations; a history of recurrent miscarriages or thromboses; and autoimmune diseases. Furthermore, participants were required to have a body mass index below 30 kg/m^2^.

### Monitor ovulation and endometrial biopsy

Based on the menstrual cycle, we advised the patient on the optimal day to use an ovulation self-test strip for monitoring luteinizing hormone levels. After receiving a positive result from the strip, the patient quickly reached out to the study coordinator for ultrasound monitoring of ovulation. Endometrial biopsies were conducted exactly six days post-ovulation. The endometrium was carefully aspirated using a specialized rotating suction tube designed for tissue collection from the endometrial cavity (Qiuheng, Y3.2, CHN). The contents of the suction tube were then transferred onto a gauze compress and separated into two portions: one portion was fixed in 4% paraformaldehyde (White Shark, CHN) for future endometrial dating analysis, while the other was promptly placed in liquid nitrogen for proteomic studies.

### Dating of endometrial samples

Samples were fixed in a solution of 4% paraformaldehyde for routine assessment. An automated staining system (Dako dyeing machine, DP260, CHN) was utilized for the application of hematoxylin and eosin (HE) stain. The determination of whether the sample belonged to the mid-luteal phase was made by two experts from the pathology department.

### Protein extraction and digestion

Proteins were extracted and digested from prepared endometrial tissue by homogenizing it in a pre-cooled solution of PBS (0.01 M) containing protease inhibitor cocktail (0.01 M) (Sigma, USA), 8 M urea, NaF (0.05 M), and Na3VO4 (0.01 M). The resulting homogenate was then centrifuged at 12,000 × g for 15 min at 4 °C to obtain the supernatant, which was transferred to a new centrifuge tube. Tris-saturated phenol (pH 7.8) in the same volume as the supernatant was added to the mixture and vortexed for 5 min before being centrifuged again at 12,000 × g for 20 min at 4 °C. Subsequently, an equal volume of Tris–HCl buffer (50 mM, pH 8.0) was added to the mixture and vortexed for another 5 min followed by centrifugation at 12,000 × g for another 20 min at 4 °C. After eliminating the upper aqueous phase, the protein content was precipitated by introducing a specific volume of 0.1 M ammonium acetate in methanol and storing it at − 20 °C overnight. The mixture underwent centrifugation for 20 min (4 °C, 12,000 × g), and the liquid above was discarded. The protein sediment settled at the bottom of the tube was rinsed twice with methanol, and the extracted proteins were freeze-dried. To reduce disulfide bonds within the proteins, a solution containing 10 mM dithiothreitol was applied to treat the protein sample for 30 min at 37 °C. Subsequently, alkylation of cysteine residues occurred by treating them with a solution containing 20 mM iodoacetamide at room temperature in darkness while constantly shaking for 45 min. Enzymatic digestion using trypsin (1:20 w/w ratio) took place under strict control conditions at 37 °C for a duration of 12 h; an additional enzyme supplementation occurred after six hours. Peptides resulting from this process were purified using C18 solid-phase extraction according to manufacturer’s guidelines (CNW, UK). Concentrations of peptides obtained post-trypsin digestion were determined utilizing BCA Protein Assay kit (Pierce Biotechnology, USA). Adjustments were made to ensure equal protein concentrations across all samples. Digestion efficiency was assessed through liquid chromatography/mass spectrometry (LC–MS) analysis conducted on Thermo Fisher Scientific equipment.

### Enrichment of phosphorylated peptides

The phosphorylated peptides were captured using TiO2 beads (GL Sciences, Japan). The TiO2 beads were activated using a solution of 1 M glycolic acid buffer A (composed of 50% acetonitrile and 2% TFA, V/V). To re-suspend the peptides, they were mixed with buffer A and combined with the activated TiO_2_ beads at a ratio of peptide to TiO_2_ beads of 1/4 w/w. This mixture was then incubated for one hour at room temperature. Subsequently, the non-phosphorylated peptides were removed by washing the TiO_2_ beads with 50% ACN. The washed TiO_2_ beads underwent two additional washes using a solution containing 20 mM ammonium acetate in 50% ACN. Elution of the phosphorylated peptides from the TiO_2_ beads was achieved by adding 200 μl of NH4OH solution at concentrations of first time: 0.3 M and second time:0.5 M respectively. Finally, after enrichment, centrifugation, concentration, and drying process, these enriched phosphorylated peptides were frozen at − 20 °C for storage purposes.

### DIA spectral acquisition and data analysis

The LC–MS system utilized for analysis consisted of a nanoACQUITY UPLC M-Class system (manufactured by Waters, USA) and a Q-Exactive HF mass spectrometer (manufactured by Thermo Fisher Scientific, USA) to obtain DIA spectral data. Each sample was initially loaded onto a C18 RP trap column (with 5 μm particle size, 100 Å pore size, and dimensions of 180 μm inner diameter × 20 mm length; manufactured by Waters, USA), followed by separation on a C18 RP analytical column (with 1.8 μm particle size and dimensions of 100 μm inner diameter × 150 mm length; manufactured by Waters, USA). The separation process involved using a linear ACN gradient with solvent B ranging from 2 to 8% over an interval of 8 min, followed by an increase from 8 to 35% over the subsequent duration of approximately two hours and employing a flow rate of 300 nl/min. Solvent A comprised primarily H_2_O with minor additions of formic acid (99.9% H_2_O, 0.1% formic acid), while solvent B consisted mainly of ACN along with small amounts of formic acid (99.9% ACN, 0.1% formic acid). Electrospray ionization into the Q-Exactive HF instrument was achieved through application of a voltage at around − 2 kV accompanied by maintaining the temperature at approximately 290 °C. The parameters set for DIA mass spectrometry were as follows: (a) full scan range: m/z values between 350 and 1200; (b) precursor ion resolution:60,000; (c) AGC target value:3e6; (d) maximum IT value:50 ms; (e) normalized collision energy during high-energy collision dissociation(HCD):27%; (f) MS2 scanning windows:34 in total with each window covering26 m/z units(there is also an overlap of 1 m/z between adjacent windows); (g) MS2 scan resolution:30,000; (h) MS2 AGC target value:1e6; (i) MS2 maximum IT:auto. The same procedure described above was employed for collecting DIA data aimed at enriching phosphorylated peptides. Finally, the Spectronaut software (version14 0.0, Biognosys, Switzerland) was used to analyze the obtained DIA data. The default parameters were configured for conducting DIA data analysis, with a protein FDR threshold set at 1%.

### Data dimensionality reduction

A total of 4,412 proteins were identified by proteome sequencing performed on six recurrent implantation failure (RIF) patients and six controls. The RIF-related datasets (GSE183837 and GSE111974) were retrieved from the Gene Expression Ontology (GEO) database (https://www.ncbi.nlm.nih.gov/geo/). The single-cell sequencing dataset GSE183837 (platform: GPL24676) was derived from RNA-seq of endometrial tissue from six RIF patients and three controls [13]. The GSE111974 dataset (platform: GPL17077) was used as a validation set and contained 24 RIF and 24 control endometrial tissue [14].

### Selection and functional enrichment analysis of differentially expressed proteins (DEPs)

Proteomics sequencing results were analyzed by principal component analysis (PCA) as well as inter-sample spearman correlation in order to ensure sample reproducibility of proteomics sequencing data. The DEPs between RIF and controls were picked out using the ‘limma’ (version 3.46.0) [15] (|log2FC|> 0.5, *p* < 0.05 [16]). Then, ‘ggplot2’ (version 3.4.1) [17] and ‘pheatmap’ (version 2.12.1) were utilized to plot volcano maps and heatmaps, respectively. DEPs converted to gene Symbol using unportID were recorded as differentially expressed genes1 (DEGs1) and immediately used for further analysis. The ‘clusterProfiler’ (version 4.0) [18, 19] was implemented for the Kyoto Encyclopedia of Genes and Genomes (KEGG) enrichment analysis and Gene Ontology (GO) annotation of DEGs1.

### Single-cell data analysis

The GSE183837 were filtered via the ‘Seurat’ (version 4.0.5) [20], and retaining genes with expression data in at least three cells and cells with a number of detected genes above 200. Next, chondrogenic were calculated using the PercentageFeatureSet function, cells with less than 20% chondrogenic were retained, and violin plots were drawn to characterize the data. Then, the data were normalized using NormalizeData. FindVariableFeatures was used to identify genes with high variability in expression between cells. These cells were then subjected to PCA. The tSNE algorithm was then performed on the cells obtained in the previous step to obtain clusters of different cells. On top of this, the marker genes [21] of each cell subpopulation were used to annotate the type of subpopulation of cells via ‘SingleR’ (version 1.831) [22]. The functions of different cell clusters were enriched for further exploration through ‘ReactomeGSA’ (version 1.12.0). Finally, different cell clusters between RIF and controls were differentially analyzed through FindMarkers (|log2FC|> 0.5, *p* < 0.05 [16].

### Analysis of cells communication

Cellular expressions of ligands and receptor pairs were monitored by Cellchat as an assessment of intercellular communication. Next, in order to investigate the mode of action between cells, non-negative matrix factorization (NMF) was used to identify patterns of communication within cells.

### Selection and functional enrichment analysis of the key genes

Differentially expressed genes 2 (DEGs2) in NK cells between RIF and controls were intersected with DEGs1 to obtain candidate genes. Afterward, the protein–protein interaction (PPI) between candidate genes was explored immediately by STRING (https://string-db.org). Then, the expression of candidate genes was observed in proteomics sequencing data and validated in GSE111974 to identify key genes. The distribution of key genes in NK cells was subsequently observed. The ‘clusterProfiler’ was implemented for the GSEA enrichment analysis of key genes to find the signaling pathways in which key genes were involved. Moreover, the signaling pathways in which the key genes co-participate were obtained by intersecting with the signaling pathways enriched by DEPs.

### Proposed temporal sequence analysis of key genes and regulatory networks

Pseudo-temporal analysis was performed on the NK cells using the Monocle algorithm (version 2.18.0) [23] in order to observe the dynamics of the cells during development. In addition, the regulatory network of transcription factors (TFs) and key genes was explored using Single-Cell Regulatory Network Inference and Clustering (SCENIC) (version 1.3.1) [24].

### Statistical analysis

The R programming language (version 4.2.2) was used for all analyses. A *p* value of less than 0.05 was deemed statistically significant unless otherwise noted.

## Results

### Identification of 1366 DEPs

**Fig. 1:**
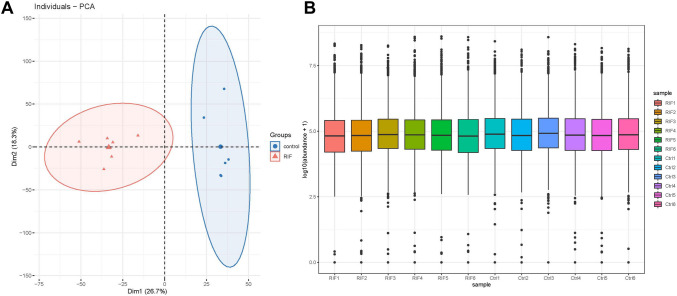
DEPs were identified in recurrent implant failure (RIF) samples and healthy control (Ctrl) samples. **A** The PCA in samples (RIF vs. Ctrl); Correlation heat map between samples. **B** Map of protein abundance in each sample

**Fig. 2:**
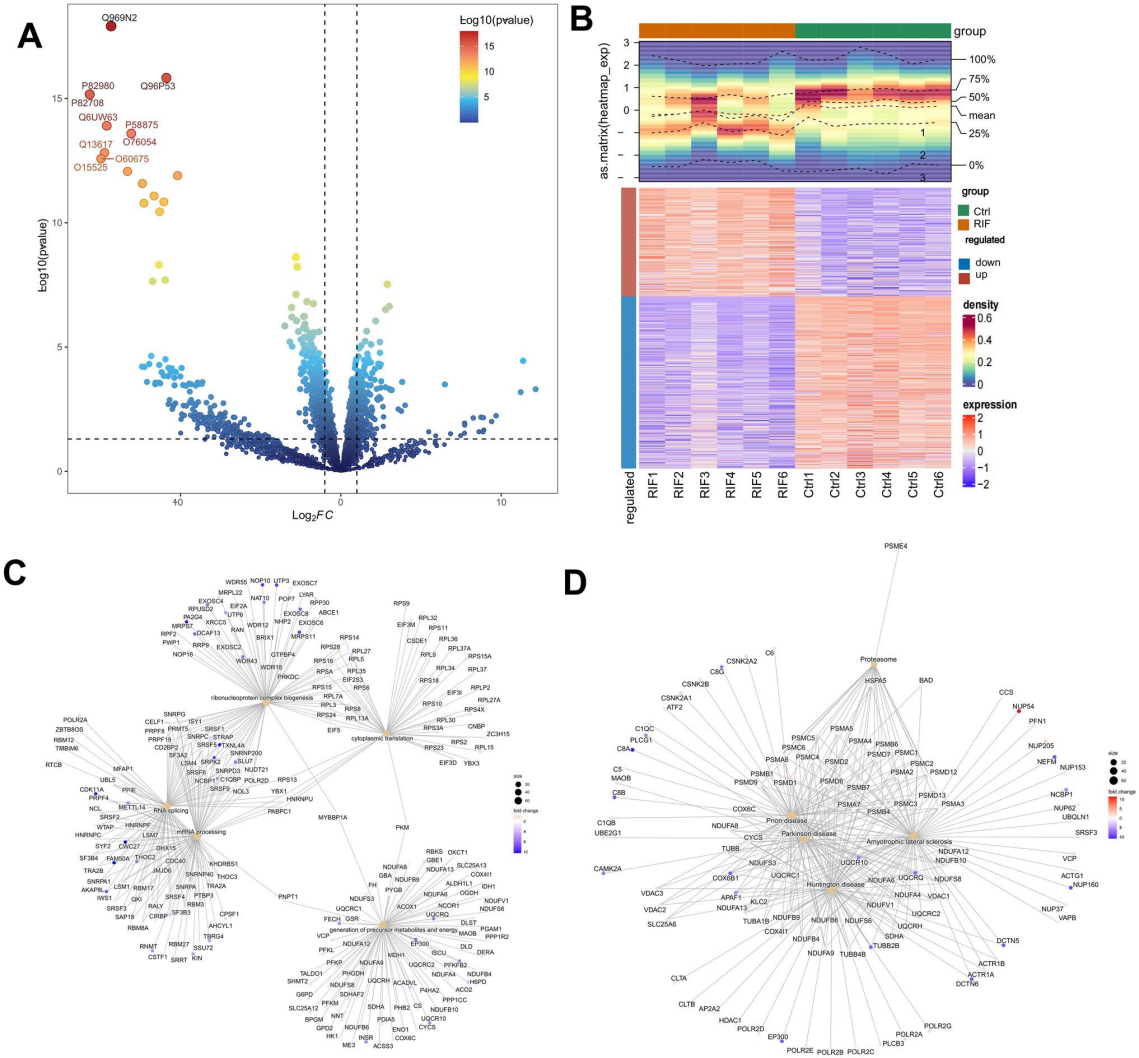
DEPs/DEGs and functional analysis. **A** Volcano map of DEP. The x-axis represents the fold change (RIF vs. Ctrl, on a logarithmic scale), and the y-axis represents -log10 (*p* value) *p* value < 0.05; **B** Heat map of DEP in the (RIF vs. Ctrl) group; **C** Gene Ontology (GO) Enrichment analysis network diagram; **D** Network map of Kyoto Encyclopedia of Genes and Genomes (KEGG) analysis

In the proteomic data, PCA analysis showed a good separation between RIF group and control group, and Spearman correlation analysis showed that the biological replicates of RIF / Ctrl had good reproducibility (Fig. 1A, B). Compared with control group, T 1366 DEPswere identified, in which 530 DEPs were up-regulated and 836 DEPs were down-regulated in the RIF (Fig. 2A). And the expression of DEPs between the different subgroups was confirmed by heatmaps (Fig. 1B). The 1210 DEGs1 transformed by unportID were analyzed for functional enrichment. GO enrichment analysis indicated DEGs1 were involved in processes, such as ‘cytoplasmic translation’, ‘biogenesis of ribonucleoprotein complexes’, and ‘mRNA process’ (Fig. 2C). KEGG enrichment analysis showed that DEGs1 mainly were enriched in ‘amyotrophic lateral sclerosis’ and ‘proteasomes’ pathways (Fig. 2D).

### Identification of cell subtypes

**Fig. 3:**
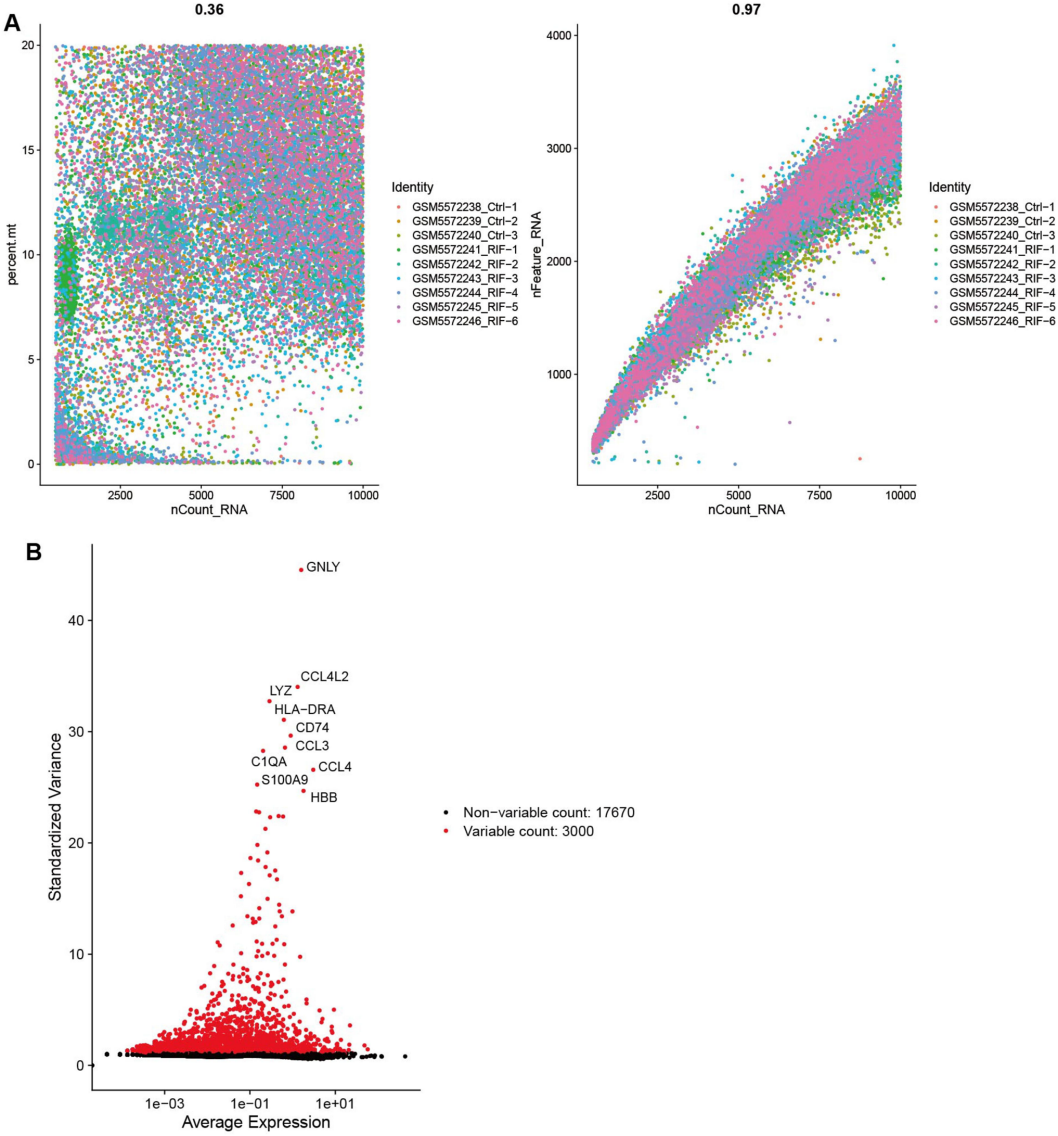
Identification of cell subtypes. **A** Sample feature scatter plot. **B** Top 10 genes between cells

In the single-cell dataset, 22,442 cells were obtained after quality control without significant outlier samples. The ten genes exhibiting the highest variability in expression across cells were selected and labeled, and no outlier samples were retained for further analysis (Fig. 3A, B).

**Fig. 4:**
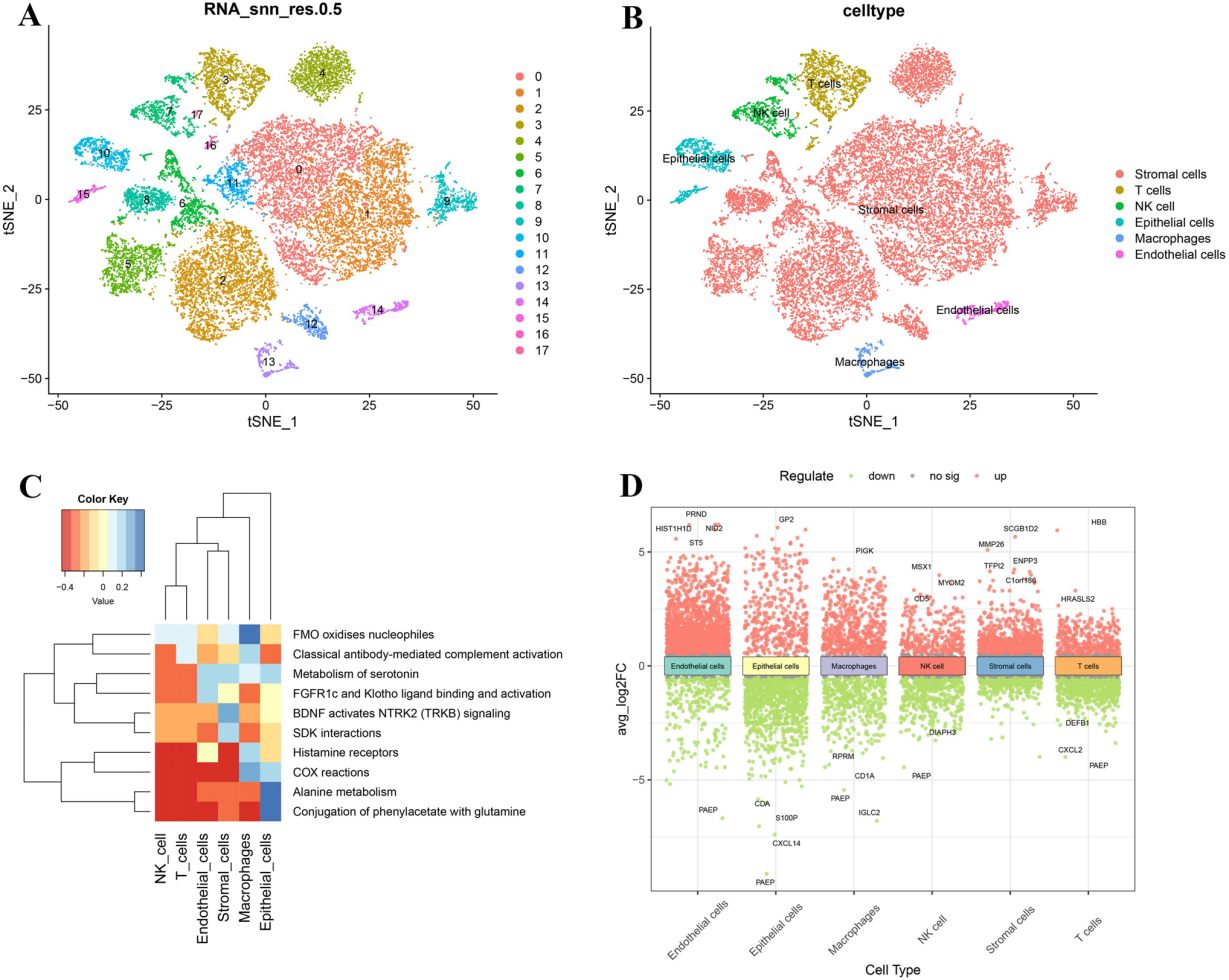
Annotate the endometrial cells. **A**, **B** Cell type tSNE distribution map. **C** Functional enrichment analysis of cell clusters. **D** Single-cell differential analysis volcano plot

The dataset was then subjected to PCA analysis to reduce dimensionality, resulting in the selection of 18 principal components. These components were used to define 18 distinct cell clusters through t-Distributed Stochastic Neighbor Embedding (tSNE) analysis (Fig. 4A). Subsequently, these clusters were annotated into six cellular groups: fibroblast-like stromal cells, epithelial cells, natural killer (NK) cells, macrophages, T cells, and endothelial cells (Fig. 4B). These clusters were annotated into six cellular groups: fibroblast-like stromal cells, epithelial cells, natural killer (NK) cells, macrophages, T cells, and endothelial cells (Fig. 4C). Furthermore, the DEGs2 in NK cells was analyzed. The analysis identified 752 DEGs, with 291 genes upregulated and 461 genes downregulated in NK cells (Fig. 4D).

**Fig. 5:**
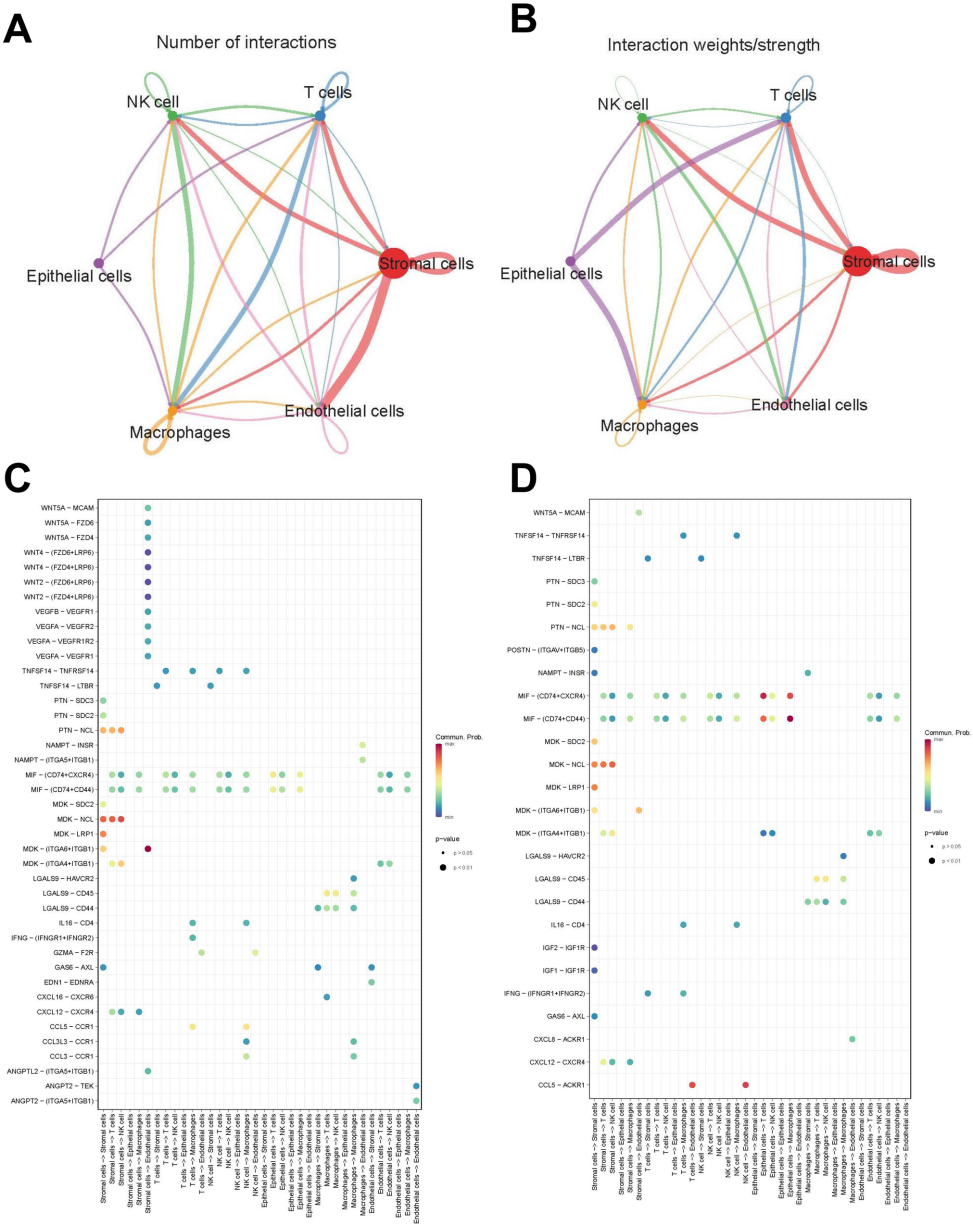
Cell–cell communication analysis. **A** Cell communication network diagram (RIF); **B** Cell communication network diagram (Ctrl). **C**, **D** Cellular interaction bubble heat map (RIF vs. Ctrl)

The analysis of the cellular communication network in RIF revealed robust interactions between fibroblast-like stromal cells and NK cells, T cells, and endothelial cells (Fig. 5A). In contrast, within the control group, fibroblast-like stromal cells exhibited stronger interactions with NK cells and T cells, while epithelial cells demonstrated enhanced interactions with T cells and macrophages (Fig. 5B). The intercellular receptor–ligand results showed a heightened presence of multiple endothelial growth factors, including VEGFA, VEGFB, and VEGFR1, as well as the Wnt signaling pathway, between fibroblast-like stromal cells and endothelial cells in RIF compared to the control group. Additionally, there was an increased presence of inflammatory factors, such as IL6-CD4, CCL5-CCR1, and CCL3-CCR1, in the interactions between NK cells and macrophages in RIF, which were not observed in the control group (Fig. 5C, D).

### Changes in cellular communication patterns between RIF and control

**Fig. 6:**
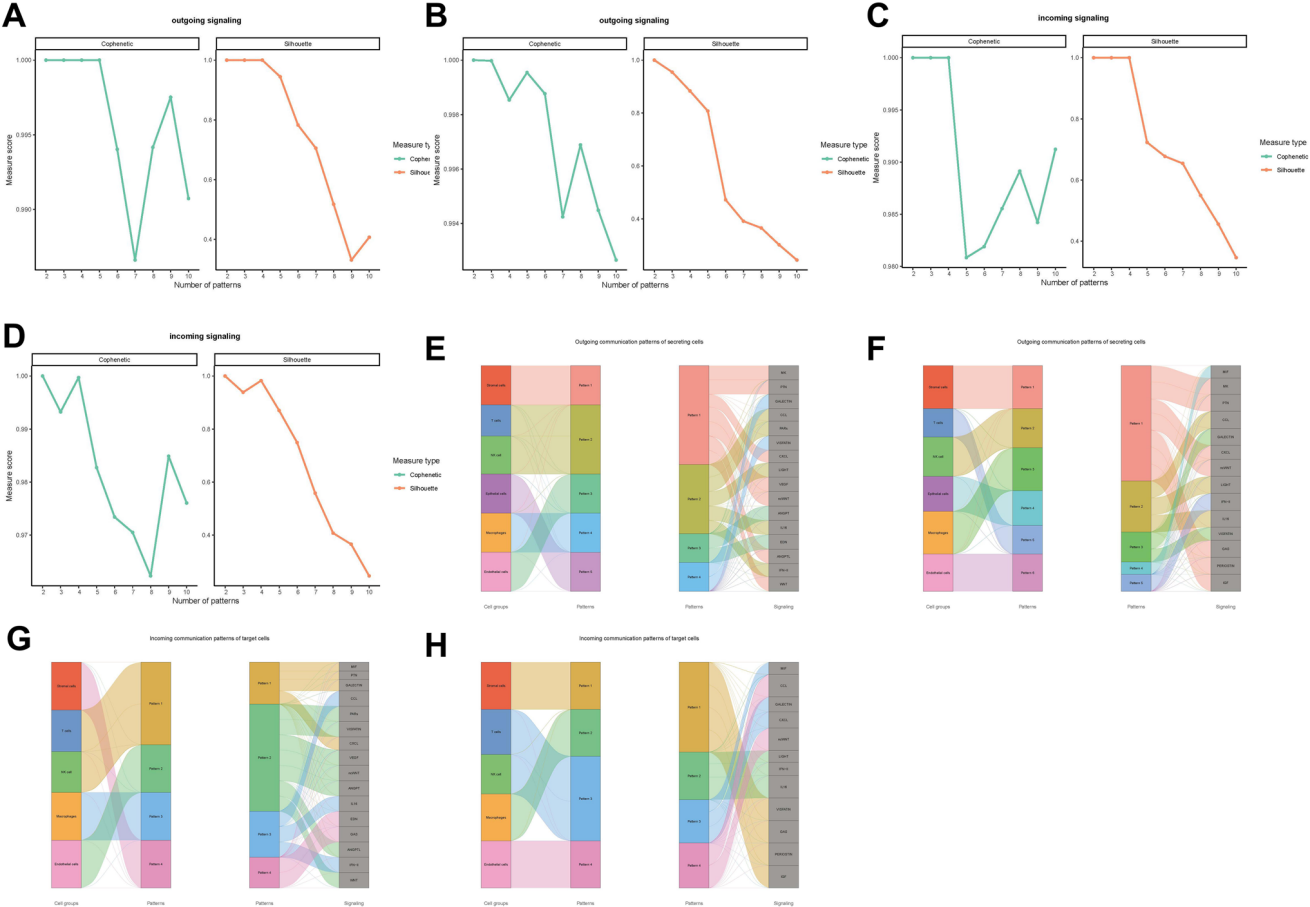
Cellular communication patterns between RIF and control. **A**, **B** Outgoing communication patterns. **C**, **D** Incoming communication patterns. **E**, **F** Sankey diagrams of different efferent patterns of cells. (G-H) Sankey diagrams of different afferent patterns of cells

Analysis of the outgoing communication patterns among cells in the RIF and control groups revealed distinct categorizations. Specifically, cells in the RIF group were classified into five distinct patterns, while those in the control group exhibited six different patterns (Fig. 6A, B). Conversely, the incoming cellular communication patterns were more uniform, with four patterns observed in both RIF and control groups (Fig. 6C, D). Subsequently, Sankey diagrams were employed to visually represent the differential efferent-afferent patterns of cells between the two groups. In the RIF group, NK cells and T cells shared similar efferent patterns and were implicated in pathways, such as pattern recognition receptors (PRRs), interferon (INF)-II, and other immune-related signaling pathways. In contrast, the control group displayed unique efferent patterns for each cell type, with NK cells also participating in immune-related signaling pathways (Fig. 6E, F). Similarly, NK and T cells in both the RIF and control groups exhibited identical afferent patterns and were associated with protein factors, including MIF, PTN, GALECTIN, and CXCL (Fig. 6G, H).

### Identification of three key genes

**Fig. 7:**
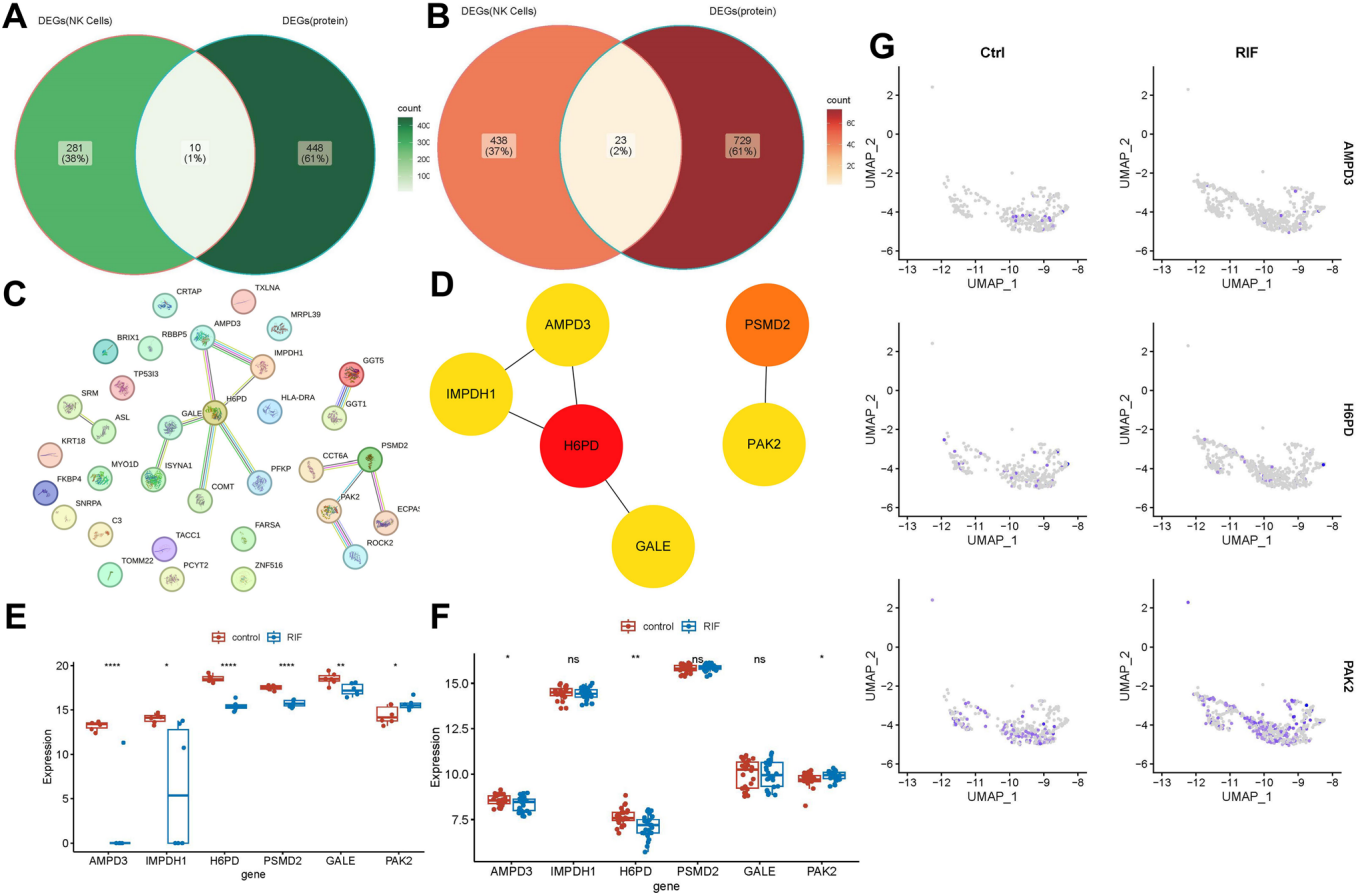
Identification of three key genes. **A**, **B** Up-regulated and down-regulated genes Venn diagram. **C**, **D** Names of 33 candidate genes and PPI network of the candidate genes. **E**, **F** Verification of key gene expression in the validation set and GSE111974 dataset. **G** Verification of key gene expression in NK cells in RIF

A total of 33 candidate genes were identified upon intersecting the ID-transformed 1,210 differentially expressed genes (DEGs1) with the 752 DEGs2 derived from natural killer (NK) cells. Among these, 10 genes were up-regulated, with 23 down-regulated in RIF group (Fig. 7A, B). Subsequently, a very robust interaction network among these 33 candidate genes was clearly demonstrated in the PPI network. The names of 33 candidate genes and PPI network are listed in the Fig. 7C, with particularly strong linkages observed between the genes *H6PD*, *AMPD3* and *IMPDH1* (Fig. 7D). The expression patterns of *AMPD3*, *H6PD*, and *PAK2* were consistently observed in both the validation cohort and the GSE111974 dataset, highlighting them as key genes of interest. Notably, AMPD3 and H6PD were up-regulated, whereas PAK2 exhibited down-regulation in RIF group (Fig. 7E, F). Finally, compared to control group, the expression of key genes in NK cells showed that AMPD3 and H6PD were reduced in NK cells in RIF, while PAK2 expression was increased, AMPD3, H6PD, and PAK2 was as crucial genes (Fig. 7G).

### Pathogenic Escherichia coli infection correlated with RIF

**Fig. 8:**
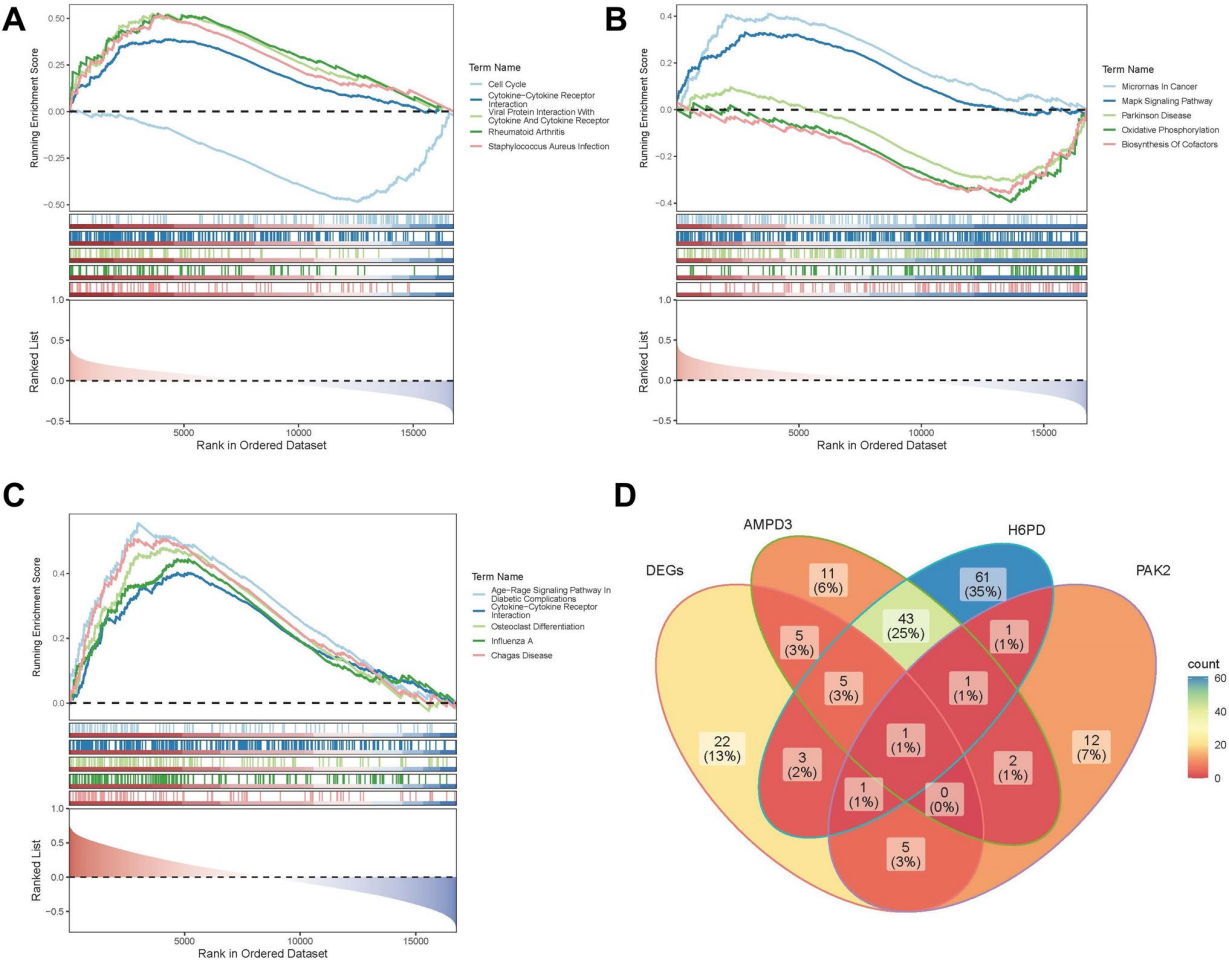
Pathogenic correlated with RIF. **A**–**C** GSEA of key genes. **D** Crossing the signaling pathways of key genes

GSEA analysis showed that among the signaling pathways associated with AMPD3, only the ‘cell cycle’ pathway was significantly inhibited (Fig. 8A). In contrast, ‘microRNAs in cancer’ and ‘Mapk signaling pathway’ were promoted in the signaling pathways associated with *PAK2* (Fig. 8B). Notably, all signaling pathways related to H6PD exhibited an activated state (Fig. 8A, B, C). Ultimately, the intersection of the signaling pathways of these key genes with those enriched for differentially expressed proteins (DEPs) revealed a potential association with ‘pathogenic *Escherichia coli* infection,’ which may be linked to RIF (Fig. 8D).

### Mechanism of key genes

**Fig. 9:**
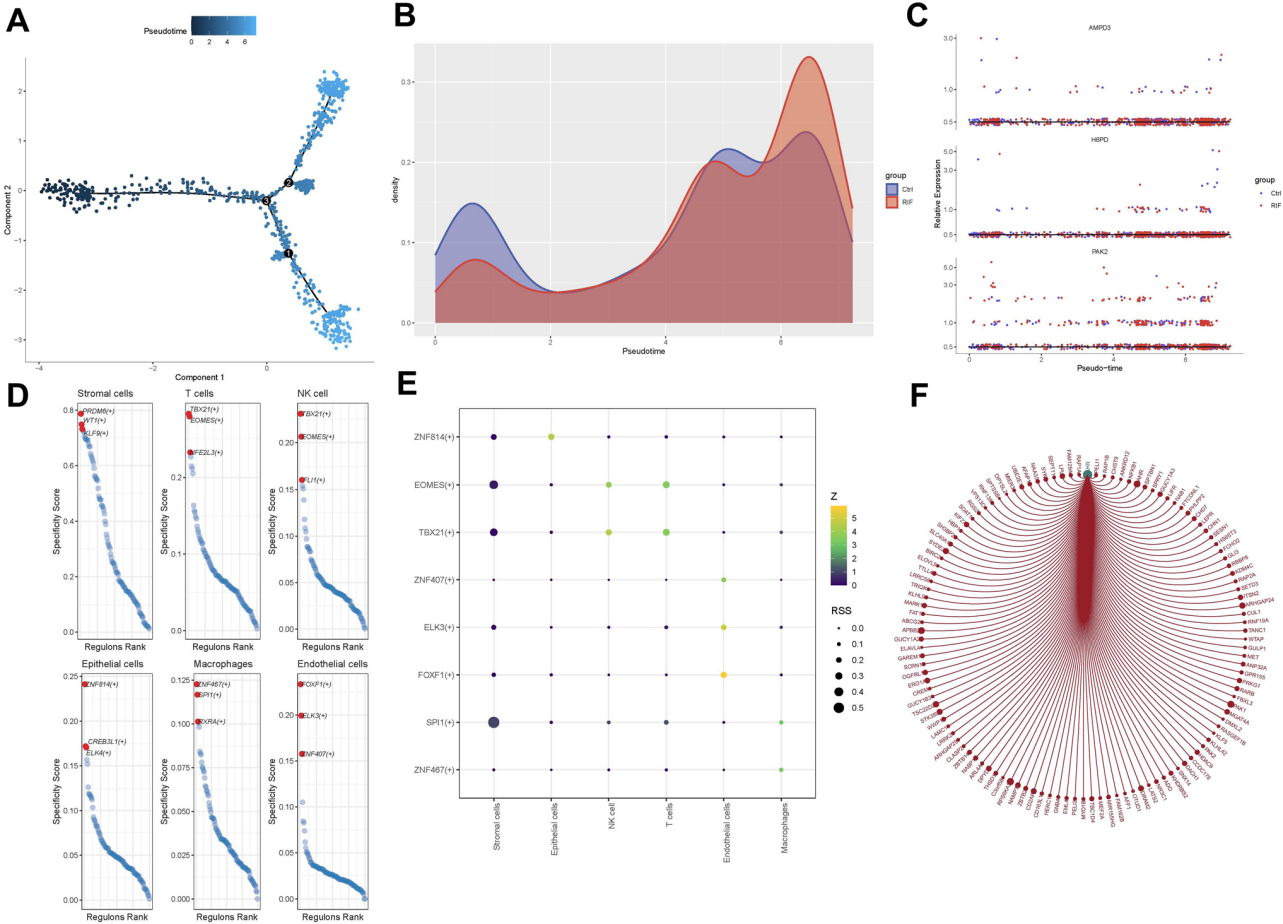
Cellular pseudo-temporal analysis and the mechanism of key genes. **A**, **B** Pseudo-temporal analysis of NK. **C** Pseudo-temporal analysis of the key genes. **D**, **E** Transcription factors in different cells. **F** Network map of TFs

The cellular pseudo-temporal analysis showed that NK cells exhibited higher density in the control group at the initial stage, whereas by the end of the observation period, a greater density of NK cells was observed in RIF group (Fig. 9A, B). In the pseudo-temporal analysis of key gene expression, *H6PD* showed significantly elevated levels in the late stages of cell differentiation in the normal group, whereas the relative expression of *PAK2* was notably higher in the RIF group (Fig. 9C). TFs varied across different cells, with *TBX21*, *EOMES*, and *FLI1* identified as key regulators in NK cells (Fig. 9D). And *EOMES* and *TBX21* demonstrated the highest specificity scores among these TFs (Fig. 9E). Finally, the network map of TFs with key genes revealed a strong regulatory relationship between *HR* and *PAK2* (Fig. 9F).

## Discussion

RIF is a critical limiting factor in enhancing the clinical pregnancy rate following IVF-ET, characterized by the inability to conceive after multiple embryo transfers. The etiology of RIF is complex and diverse, presenting significant diagnostic and therapeutic challenges for patients with infertility. Currently, there is no entirely effective method to detect RIF. Therefore, there is an urgent need to explore potential therapeutic targets and effective treatments for RIF. Embryo implantation is a complex process that involves maternal immune tolerance, and the establishment of maternal–fetal crosstalk is essential for a successful pregnancy [25]. Numerous studies have elucidated the diverse impacts of immune cells within the endometrium on key processes, such as embryo implantation, trophoblast invasion, vascular remodeling, and the maintenance of immune tolerance [7]. Specifically, NK cells, as key immune cells in the female reproductive system, are involved in anti-infection immunity during pregnancy, the invasive behavior of trophoblast cells, and the remodeling process of uterine spiral arteries [26], which are crucial for maintaining a normal pregnancy. This study utilized proteomic and single-cell transcriptomic data to identify key genes (*AMPD3, H6PD,* and *PAK2*) in RIF through a series of bioinformatics analyses and investigated their potential mechanisms of action. This provides new insights for the diagnosis and treatment of RIF.

Quality control and cell annotation were performed on the single-cell dataset, identifying several cell types in the endometrium, including fibroblast-like stromal cells, NK cells, T cells, and endothelial cells. Previous studies suggest that endometrial NK cells (u-NK) are less cytotoxic compared to peripheral blood NK cells (p-NK) [27]. These NK cells serve dual roles: on the one hand, they function as immune cells, contributing to anti-infectious immunity during pregnancy; on the other hand, they participate in the regulation of trophoblast invasion and uterine artery remodeling, which are essential for the maintenance of normal pregnancy [28]. Given the significant role of NK cells in these processes, we have selected them as the focus of our study. The cellular pseudo-temporal analysis showed that NK cells were initially denser in the control group, while at the end of the period, a greater density of NK cells was observed in the RIF group. Current research suggests that CD57 + cells represent the terminal stage of NK cells, and the expression of CD57 in NK cells is associated with the expression of granzyme A, granzyme B, and perforin, indicating that CD57 may be a marker of high cytotoxic potential [29, 30]. An increase in the number of CD57 + NK cells was found in the decidua of spontaneous abortions, and these cells can activate local cytokines to attack the trophoblast, leading to pregnancy failure [31]. Both findings support our results above.

The pseudo-temporal trends of key genes *AMPD3, H6PD,* and *PAK2* from normal to disease state are consistent with proteomic analysis.

Adenosine monophosphate deaminase (AMPD) is a protein-coding gene that catalyzes the deamination reaction in nucleotide metabolism. AMPD3 is one of the three members of this family. AMPD3 is expressed in various tissues including muscle and placenta. [32]. AMPD3 exhibits evidence associated with high-density lipoprotein cholesterol (HDL-C) [33]. Dyslipidemia is not only closely related to pregnancy complications, but also contributes to pregnant women who receive ART reproductive outcomes adverse effects [34].

Hexose-6-phosphate dehydrogenase (H6PD) is a protein-coding gene located in the endoplasmic reticulum of various cells. It catalyzes the first two steps of the pentose phosphate pathway by converting 6-phosphogluconate and NADP into NADPH and 6-phosphogluconate. A close relationship between H6PD and conditions, such as Polycystic Ovary Syndrome (PCOS) and insulin resistance [35]. This suggests that H6PD may be a key gene that plays a role in RIF.

The p21-activated kinases (PAKs) are serine/threonine protein kinases. Studies have shown that the role of PAK2 in PCOS is closely related to its impact on OS [36]. Previous research indicated that PAK2 gene knockdown significantly reduced the formation rate of early embryonic blastocysts. Furthermore, the reduction of PAK2 leads to a significant increase in abnormal spindle assembly and chromosomal abnormalities in embryos [37]. When PAK2 is knocked down in embryos, the production of reactive oxygen species (ROS) increases. Studies have indicated that by eliminating the excessive accumulation of ROS, the poor decidualization caused by downregulated Sirtuin1 (SIRT1) expression in RIF patients can be improved [38]. These findings suggest that ROS plays a significant role in the occurrence and development of RIF and is closely related to the content of PAK2 and ROS. This implies that PAK2 could be a key gene for the diagnosis and treatment of RIF.

**Fig. 10:**
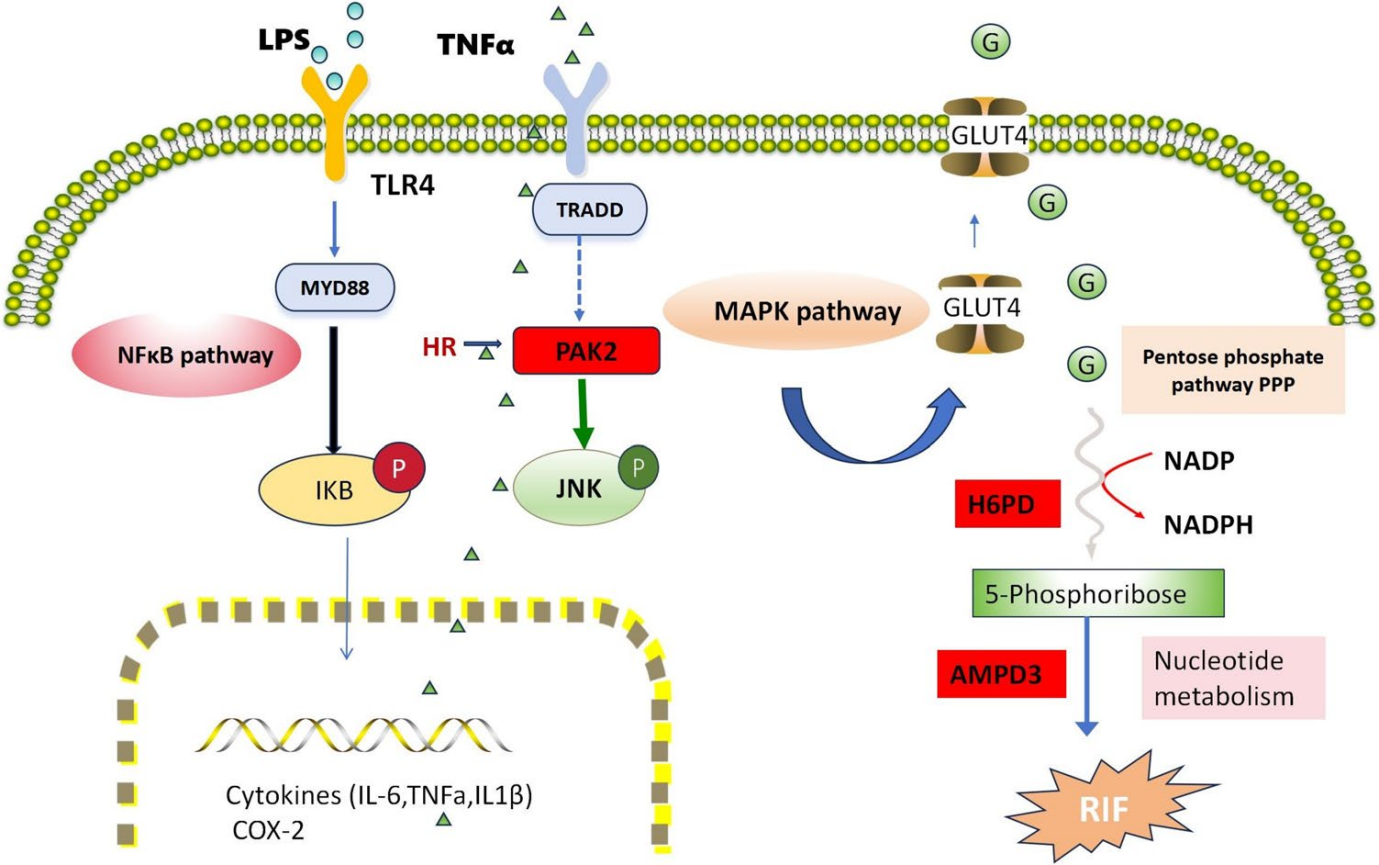
Predictive pathways of key genes in RIF

This study conducted a cross-analysis of the signaling pathways associated with the three key genes and those enriched by DEPs. A significant correlation was identified between the ‘pathogenic Escherichia coli infection’ pathway and RIF. This finding implicates a potential role for NK cells in mediating the link between infection and failure of embryo implantation. Cicinelli et al*.* [39] were the first to detect *Streptococcus*, *Staphylococcus*, *Enterococcus faecalis*, *Escherichia coli*, and mycoplasma in the endometrium of women with chronic endometritis (CE) through microbial culture. Numerous studies have also indicated that infections can cause changes in the physiological state and function of the endometrium, leading to shifts in the implantation window or abnormal uterine motility, ultimately resulting in adverse outcomes, such as recurrent implantation failure, spontaneous abortion, and infertility [40]. During Gram-negative infections, the lipopolysaccharides (LPS) from the outer wall of bacterial cell walls induce the production of selectin E, CXCL1, CXCL13, and other cytokines by endometrial microvascular endothelial cells in this unique microenvironment [41]. B lymphocytes invade the functional layer of the endometrium and even enter the epithelium, with some of the accumulated B lymphocytes differentiating into plasma cells [42]. Infections also affect endocrine signal transduction and alter the endometrium’s response to sex hormones. It has been reported that infections affect insulin signal transduction and metabolic endocrine control [43], and in rats with endometritis induced by Escherichia coli and Staphylococcus aureus, the activity of iNOS and the levels of NO and COX-2 are elevated [44, 45]. Utilizing ReactomeGSA enrichment analysis, the present study identified a significant correlation between NK cells, which are key players in the immune response, and several functional pathways, notably the COX response. Additionally, an increased presence of inflammatory factors, such as IL6-CD4, CCL5-CCR1, and CCL3-CCR1, was observed in the interactions between NK cells and macrophages within the study group, in contrast to the control group, which lacked these factors. These observations suggest a potential role for NK cells in the RIF associated with infection. This role may be mediated by cyclooxygenase-2 (COX-2), the rate-limiting enzyme in the production of prostaglandin E2 (PGE2), a compound with established immunomodulatory properties (Murakami and Kudo, 2004). Other studies have shown that the use of COX-2 inhibitors can prevent pregnancy problems caused by LPS, such as changes in the DBA + uNK cell subset and embryonic loss. The role of COX-2 in RIF is also expected to become a new research direction [46]. Nevertheless, the transcription factor regulatory network uncovered in this study offers profound insights into the molecular underpinnings of RIF. Our findings indicate that transcription factors, including *TBX21, EOMES,* and *FLI1* within NK cells, are pivotal in the regulation of key gene expression. Notably, HR was identified as a potent regulator of PAK2, underscoring the intricate interplay between transcription factors and critical genes in the context of RIF. The mechanisms by which the three key genes—AMPD3, H6PD, and PAK2—influence RIF via metabolic pathways, particularly in the context of Escherichia coli infection, remain to be fully elucidated. Studies have found that Escherichia coli increases inflammatory factors, such as iNOS, IL-1β, TNF-α, IL-6, etc. [45], through the NF-κB signaling pathway, leading to endometritis [47]. This recognition is carried out through the TLR4 and MyD88-dependent cell signaling pathway under the action of Escherichia coli LPS. In addition, the inflammatory factor TNF-α can cause tissue damage through the MAPK pathway. After MAPK activation, it stimulates the translocation of glucose transporter type 4 (GLUT4) to the endometrium, thereby actively promoting the increase in glucose uptake in the tissue, thus producing ATP through glycolysis. The pentose phosphate pathway produces 5-Phosphoribose, providing a substrate for nucleotide metabolism. In the present study, the three key genes under investigation—PAK2, H6PD, and AMPK—are implicated in the regulation of the MAPK signaling pathway, the pentose phosphate pathway, and nucleotide metabolism, respectively. By integrating the findings of this study with existing literature, we have delineated the potential metabolic pathways through which these genes exert their influence (Fig. 10).

In this study, we successfully identified three key genes (AMPD3, H6PD, and PAK2) associated with RIF in the endometrium. These genes are implicated in diverse biological processes and may serve as potential targets for the diagnosis and treatment of RIF. But the precise functional roles of these genes and their interactions with other molecules still require further elucidation. Future studies are warranted to conduct additional validation in larger cohorts to strengthen our findings.

Although this study has yielded some meaningful findings, there are still certain limitations. To achieve a deeper understanding and validation of the findings in this study, future research could consider expanding the sample size to include participants with more diverse backgrounds and characteristics. This approach would enhance the reliability and the applicability of the results, thereby providing more comprehensive and robust evidence to support related fields.

## Supplementary Information

Below is the link to the electronic supplementary material.

Supplementary file1 (DOCX 17 KB)

## Data Availability

No datasets were generated or analysed during the current study.

## References

[1] Benkhalifa M, Joao F, Duval C et al (2022) Endometrium immunomodulation to prevent recurrent implantation failure in assisted reproductive technology. Int J Mol Sci. 10.3390/ijms232112787 36361577 10.3390/ijms232112787 PMC9654171

[2] Arian SE, Hessami K, Khatibi A, To AK, Shamshirsaz AA, Gibbons W (2023) Endometrial receptivity array before frozen embryo transfer cycles: a systematic review and meta-analysis. Fertil Steril 119(2):229–238 36414088 10.1016/j.fertnstert.2022.11.012

[3] Coughlan C, Ledger W, Wang Q et al (2014) Recurrent implantation failure: definition and management. Reprod Biomed Online 28(1):14–38 24269084 10.1016/j.rbmo.2013.08.011

[4] Kouvidi E, Zachaki S, Tsarouha H et al (2021) Female reproductive ageing and chromosomal abnormalities in a large series of women undergoing IVF. Cytogenet Genome Res 161(12):551–555 35051945 10.1159/000521655

[5] Wei D, Liu J-Y, Sun Y et al (2019) Frozen versus fresh single blastocyst transfer in ovulatory women: a multicentre, randomised controlled trial. The Lancet 393(10178):1310–1318 10.1016/S0140-6736(18)32843-5 30827784

[6] Bellver J, Simón C (2018) Implantation failure of endometrial origin: what is new? Curr Opin Obstet Gynecol 30(4):229–236 29889670 10.1097/GCO.0000000000000468

[7] Yang F, Zheng Q, Jin L (2019) dynamic function and composition changes of immune cells during normal and pathological pregnancy at the maternal-fetal interface. Front Immunol. 10.3389/fimmu.2019.02317 31681264 10.3389/fimmu.2019.02317 PMC6813251

[8] Rao VA, Kurian NK, Rao KA (2023) Cytokines, NK cells and regulatory T cell functions in normal pregnancy and reproductive failures. Am J Reprod Immunol 89(2):e13667 36480305 10.1111/aji.13667

[9] Zitti B, Bryceson YT (2018) Natural killer cells in inflammation and autoimmunity. Cytokine Growth Factor Rev 42:37–46 30122459 10.1016/j.cytogfr.2018.08.001

[10] Rozanova S, Barkovits K, Nikolov M, Schmidt C, Urlaub H, Marcus K (2021) Quantitative mass spectrometry-based proteomics: an overview. Methods Mol Biol 2228:85–116 33950486 10.1007/978-1-0716-1024-4_8

[11] Chen Y, Song J, Ruan Q et al (2021) Single-cell sequencing methodologies: from transcriptome to multi-dimensional measurement. Small Methods 5(6):e2100111 34927917 10.1002/smtd.202100111

[12] Slovin S, Carissimo A, Panariello F et al (2021) Single-cell RNA sequencing analysis: a step-by-step overview. Methods Mol Biol 2284:343–365 33835452 10.1007/978-1-0716-1307-8_19

[13] Lai Z-Z, Wang Y, Zhou W-J et al (2022) Single-cell transcriptome profiling of the human endometrium of patients with recurrent implantation failure. Theranostics 12(15):6527–6547 36185612 10.7150/thno.74053 PMC9516226

[14] Bastu E, Demiral I, Gunel T et al (2019) Potential marker pathways in the endometrium that may cause recurrent implantation failure. Reprod Sci 26(7):879–890 30081718 10.1177/1933719118792104

[15] Ritchie ME, Phipson B, Wu D et al (2015) limma powers differential expression analyses for RNA-sequencing and microarray studies. Nucleic Acids Res 43(7):e47-e 25605792 10.1093/nar/gkv007 PMC4402510

[16] He Z, Jiang Q, Li F, Chen M, Xu Y (2021) Crosstalk between venous thromboembolism and periodontal diseases: a bioinformatics analysis. Dis Markers 2021:1–16 10.1155/2021/1776567 PMC8683231 34925639

[17] Ito K, Murphy D (2013) Application of ggplot2 to pharmacometric graphics. CPT: Pharmacomet Syst Pharmacol 2(10):1–16 10.1038/psp.2013.56 PMC3817376 24132163

[18] Wu T, Hu E, Xu S et al (2021) clusterProfiler 4.0: a universal enrichment tool for interpreting omics data. Innovation. 10.1016/j.xinn.2021.100141 34557778 10.1016/j.xinn.2021.100141 PMC8454663

[19] Ren Z, He Y, Yang Q et al (2022) A comprehensive analysis of the glutathione peroxidase 8 (GPX8) in human cancer. Front Oncol. 10.3389/fonc.2022.812811 35402257 10.3389/fonc.2022.812811 PMC8991916

[20] Hao Y, Hao S, Andersen-Nissen E et al (2021) Integrated analysis of multimodal single-cell data. Cell 184(13):3573–87.e29 34062119 10.1016/j.cell.2021.04.048 PMC8238499

[21] Shi J-W, Lai Z-Z, Yang H-L et al (2022) An IGF1-expressing endometrial stromal cell population is associated with human decidualization. BMC Biol. 10.1186/s12915-022-01483-0 36482461 10.1186/s12915-022-01483-0 PMC9733393

[22] Aran D, Looney AP, Liu L et al (2019) Reference-based analysis of lung single-cell sequencing reveals a transitional profibrotic macrophage. Nat Immunol 20(2):163–172 30643263 10.1038/s41590-018-0276-y PMC6340744

[23] Trapnell C, Cacchiarelli D, Grimsby J et al (2014) The dynamics and regulators of cell fate decisions are revealed by pseudotemporal ordering of single cells. Nat Biotechnol 32(4):381–386 24658644 10.1038/nbt.2859 PMC4122333

[24] Aibar S, González-Blas CB, Moerman T et al (2017) SCENIC: single-cell regulatory network inference and clustering. Nat Methods 14(11):1083–1086 28991892 10.1038/nmeth.4463 PMC5937676

[25] Zhong J, Li J, Burton GJ et al (2024) The functional roles of protein glycosylation in human maternal–fetal crosstalk. Hum Reprod Update 30(1):81–108 37699855 10.1093/humupd/dmad024

[26] Fu B, Zhou Y, Ni X et al (2017) Natural killer cells promote fetal development through the secretion of growth-promoting factors. Immunity 47(6):1100–13.e6 29262349 10.1016/j.immuni.2017.11.018

[27] Hernan D, Kopcow DSJA, Chen XI et al (2005) Human decidual NK cells form immature activating synapses and are not cytotoxic. Proc Natl Acad Sci U S A 102:15563–8 16230631 10.1073/pnas.0507835102 PMC1266146

[28] Yeh C-C, Chao K-C, Huang SJ (2013) Innate immunity, decidual cells, and preeclampsia. Reprod Sci 20(4):339–353 22814099 10.1177/1933719112450330 PMC3823393

[29] Abel AM, Yang C, Thakar MS, Malarkannan S (2018) Natural killer cells: development, maturation, and clinical utilization. Front Immunol. 10.3389/fimmu.2018.01869 30150991 10.3389/fimmu.2018.01869 PMC6099181

[30] Guo W, Fang L, Li B et al (2017) Decreased human leukocyte Antigen-G expression by miR-133a contributes to impairment of proinvasion and proangiogenesis functions of decidual NK cells. Front Immunol. 10.3389/fimmu.2017.00741 28702027 10.3389/fimmu.2017.00741 PMC5487407

[31] Lopez-Vergès S, Milush JM, Pandey S et al (2010) CD57 defines a functionally distinct population of mature NK cells in the human CD56dimCD16+ NK-cell subset. Blood 116(19):3865–3874 20733159 10.1182/blood-2010-04-282301 PMC2981540

[32] Miller SG, Hafen PS, Law AS et al (2021) AMP deamination is sufficient to replicate an atrophy-like metabolic phenotype in skeletal muscle. Metabolism. 10.1016/j.metabol.2021.154864 34400216 10.1016/j.metabol.2021.154864 PMC8453098

[33] Dong W, Wong KHY, Liu Y et al (2022) Whole-exome sequencing reveals damaging gene variants associated with hypoalphalipoproteinemia. J Lipid Res. 10.3389/fendo.2022.915424 35460704 10.1016/j.jlr.2022.100209 PMC9126845

[34] Jiang X, Lu X, Cai M, Liu Y, Guo Y (2022) Impact of dyslipidemia on the cumulative pregnancy outcomes after first ovarian stimulation. Front Endocrinol. 10.1371/journal.pone.0140326 10.3389/fendo.2022.915424 PMC9395644 36017313

[35] Baek K-H, Ju R, Wu W et al (2015) Association analysis between the polymorphisms of HSD11B1 and H6PD and risk of polycystic ovary syndrome in chinese population. Plos One. 10.1371/journal.pone.0140326 26452272 10.1371/journal.pone.0140326 PMC4599835

[36] Hui M, Hu S, Ye L, Zhang M, Jing X, Hong Y (2023) PAK2/beta-catenin/c-Myc/PKM2 signal transduction suppresses ovarian granulosa cell apoptosis in polycystic ovary syndrome. Biochem Biophys Res Commun 677:54–62 37549602 10.1016/j.bbrc.2023.08.004

[37] Zeng J, Liu N, Yang Y et al (2021) Pak2 reduction induces a failure of early embryonic development in mice. Reprod Biol Endocrinol. 10.1186/s12958-021-00865-3 34879863 10.1186/s12958-021-00865-3 PMC8656077

[38] Li J, Qi J, Yao G et al (2021) Deficiency of sirtuin 1 impedes endometrial decidualization in recurrent implantation failure patients. Front Cell Develop Biol. 10.3389/fcell.2021.598364 10.3389/fcell.2021.598364 PMC7876093 33585475

[39] Cicinelli E, De Ziegler D, Nicoletti R et al (2008) Chronic endometritis: correlation among hysteroscopic, histologic, and bacteriologic findings in a prospective trial with 2190 consecutive office hysteroscopies. Fertil Steril 89(3):677–684 17531993 10.1016/j.fertnstert.2007.03.074

[40] Lindsay CV, Potter JA, Grimshaw AA, Abrahams VM, Tong M (2023) Endometrial responses to bacterial and viral infection: a scoping review. Hum Reprod Update 29(5):675–693 37290428 10.1093/humupd/dmad013 PMC10477945

[41] Kitaya K, Yasuo T (2010) Aberrant expression of selectin E, CXCL1, and CXCL13 in chronic endometritis. Mod Pathol 23(8):1136–1146 20495539 10.1038/modpathol.2010.98

[42] Raja KRM, Kovarova L, Hajek R (2010) Review of phenotypic markers used in flow cytometric analysis of MGUS and MM, and applicability of flow cytometry in other plasma cell disorders. Br J Haematol 149(3):334–351 20201947 10.1111/j.1365-2141.2010.08121.x

[43] Wensveen FM, Šestan M, Turk Wensveen T, Polić B (2019) ‘Beauty and the beast’ in infection: how immune–endocrine interactions regulate systemic metabolism in the context of infection. Eur J Immunol 49(7):982–995 31106860 10.1002/eji.201847895

[44] Tiwari A, Singh P, Jaitley P et al (2018) Eucalyptus robusta leaves methanolic extract suppresses inflammatory mediators by specifically targeting TLR4/TLR9, MPO, COX2, iNOS and inflammatory cytokines in experimentally-induced endometritis in rats. J Ethnopharmacol 213:149–158 29104078 10.1016/j.jep.2017.10.035

[45] Miao Y, Ishfaq M, Liu Y et al (2021) Baicalin attenuates endometritis in a rabbit model induced by infection with Escherichia coli and Staphylococcus aureus via NF-κB and JNK signaling pathways. Domestic Animal Endocrinol. 10.1016/j.domaniend.2020.106508 10.1016/j.domaniend.2020.106508 32861957

[46] Zavan B, De Almeida EM, Salles ÉdSL, do Amarante-Paffaro AM, Paffaro VA (2016) COX-2 plays a role in angiogenic DBA+ uNK cell subsets activation and pregnancy protection in LPS-exposed mice. Placenta 44:34–45 27452436 10.1016/j.placenta.2016.06.006

[47] Fu K, Lv X, Li W et al (2015) Berberine hydrochloride attenuates lipopolysaccharide-induced endometritis in mice by suppressing activation of NF-κB signal pathway. Int Immunopharmacol 24(1):128–132 25479718 10.1016/j.intimp.2014.11.002

