# Supplementary material for: Using proteomics and single-cell sequencing to analyze the pathogenesis of recurrent implantation failure associated with uterine natural killer cells
Source: Arch Gynecol Obstet. 2025 Jun 13;312(3):885–900. doi: 10.1007/s00404-025-08074-8 (PMC12374899; doi:10.1007/s00404-025-08074-8)
Supplement: Supplementary file 1 — Supplementary file1 (DOCX 17 KB) [file 404_2025_8074_MOESM1_ESM.docx]

Supplementary table 1 Baseline characteristics

| Group | Age (y) | BMI  (kg/m2) | Times of  embryo transfers | Biochemical pregnancy yes or not | Infertility reasons |
| --- | --- | --- | --- | --- | --- |
| RIF | 21 | 24.0 | 3 | N | MOFT |
| RIF | 30 | 20.3 | 4 | N | MOFT |
| RIF | 32 | 21.5 | 3 | N | MOFT |
| RIF | 30 | 21.2 | 3 | N | MOFT |
| RIF | 33 | 26.4 | 3 | N | MOFT |
| RIF | 26 | 20.0 | 4 | N | Unknown reason |
| Con | 32 | 26.1 | 1 | Y | MOFT |
| Con | 31 | 24.3 | 2 | Y | MOFT |
| Con | 28 | 23.1 | 1 | Y | MOFT |
| Con | 28 | 24.5 | 1 | Y | MOFT |
| Con | 32 | 22.0 | 1 | Y | MOFT |
| Con | 34 | 18.3 | 1 | Y | MF |

*RIF* Recurrent implantation failure, *Con* Control, *BMI* Body mass index, *MOFT* Mechanical obstruction of the fallopian tube, *MF* Infertility due to male factors
